# The Relationship between *MALAT1* Polymorphism rs3200401 C > T and the Risk of Overall Cancer: A Meta-Analysis

**DOI:** 10.3390/medicina58020176

**Published:** 2022-01-25

**Authors:** Keming Li, Zhuo Han, Jinyu Wu, Hua Ye, Guiying Sun, Jianxiang Shi, Jianying Zhang, Peng Wang

**Affiliations:** 1 Department of Epidemiology and Statistics, College of Public Health, Zhengzhou University, Zhengzhou 450001, China; a635552617@163.com (K.L.); 15703410928@163.com (Z.H.); 18220193940@163.com (J.W.); yehua@zzu.edu.cn (H.Y.); guiyingsun18@163.com (G.S.); jianyingzhang@hotmail.com (J.Z.); 2 Henan Key Laboratory of Tumor Epidemiology, Zhengzhou University, Zhengzhou 450001, China; jianxiangshi@zzu.edu.cn; 3 Henan Institute of Medical and Pharmaceutical Sciences, Zhengzhou University, Zhengzhou 450001, China

**Keywords:** *MALAT1*, rs3200401 C > T, meta-analysis, cancer risk, colorectal cancer

## Abstract

*Background and Objectives*: At present, the association between the long non-coding RNA (lncRNA) metastasis-associated lung adenocarcinoma transcript 1 (*MALAT1*) polymorphism rs3200401 C > T and cancer risk remain controversial. The aim of this meta-analysis was to assess the association between rs3200401 C > T and cancer susceptibility. *Materials and Methods*: The databases of PubMed, EMBASE and Web of Science were searched for literature published in English until 1 September 2021. The odd ratios (ORs) and 95% confidence intervals (CIs) were applied to evaluate the strength of association in five genetic models. Heterogeneity was assessed using the Q-test and I^2^ test. Begg’s funnel plot and Egger’s linear regression test were conducted to assess publication bias. Meta-regression analysis was used to explore potential sources of heterogeneity. Trial sequential analysis (TSA) was performed to validate the reliability of the results. *Results*: A total of 10 case–control studies involving 6630 cases and 7457 controls were included in this study. The pooled ORs showed no significant association between *MALAT1* rs3200401 C > T and cancer risk in five genetic models. Similarly, the association was not found in the subgroups of control source, ethnicity and study quality. In the cancer type subgroup, the results demonstrated that the T allele increased the risk of colorectal cancer (CRC) compared with the C allele. (C vs. T: OR, 1.16; 95% CI, 1.01–1.33). *Conclusion*: In the current meta-analysis, we found no significant association between *MALAT1* polymorphism rs3200401 C > T and overall cancer risk. However, the rs3200401 C > T may be linked to a higher risk of CRC, which needs more studies to be further confirmed.

## 1. Introduction

In recent years, cancer incidence and mortality rates have increased rapidly worldwide. In 2020, there were 19.29 million new cancer cases and 9.96 million cancer deaths worldwide [1]. Despite the recent advances in medical technology for cancer, the burden of disease caused by cancer is still serious [2,3]. Therefore, it is necessary to explore the risk factors of cancer to identify and protect the high-risk population. In addition to some well-known environmental risk factors, a growing number of studies have confirmed that genetic risk factors play an important role in carcinogenesis [4,5].

Long non-coding RNAs (lncRNA), a type of RNA with a length of more than 200 bp, lack functional open reading frames (ORFs) and protein-coding capabilities [6]. They have been involved in the regulation of a variety of cellular processes, including the regulation of transcription and post-transcriptional levels, and the modification of chromatin, which are considered to be closely related with disease, especially cancer [7]. Metastasis-associated lung adenocarcinoma transcript 1 (*MALAT1*) is a nuclear-retained lncRNA, more than 8000 nucleotides in length, located on chromosome 11q13 [8,9]. *MALAT1*, which has been found abnormally overexpressed in multiple cancer tissues, is involved in cell cycle regulation, and regulates alternative splicing, pre-mRNA splicing and interacts with miRNA [10]. Thus, *MALAT1* promotes the progression, invasion and metastasis of cancer to a certain extent [11,12]. Recent studies have shown that *MALAT1* enhances the expression of serine-rich arginine splicing factor 1 (SRSF1) and activates the mammalian rapamycin target (mTOR) signaling pathway to promote the formation of gastric cancer (GC) and hepatocellular carcinoma (HCC) [13,14]. Additionally, the knockdown of *MALAT1* inhibits epithelial–mesenchymal transition and induces apoptosis in tumor cells [15,16].

The presence of single nucleotide polymorphisms (SNP) may directly or indirectly influence lncRNA expression levels to regulate the occurrence and development of cancers [17]. SNPs have been extensively studied as biomarkers, and the association between *MALAT1* polymorphism rs3200401 C > T and cancer risk has been investigated in recent years. Peng et al. showed that females with a CT genotype of rs3200401 had a lower risk of breast cancer (BC) [18]. Similarly, the subjects with TT genotype were associated with an increased risk of oral squamous cell carcinoma (OSCC) after adjusting for other variables [19]. There is no significant association between rs3200401 C > T and HCC [20,21]. Qu et al. found that rs3200401 C > T in the *MALAT1* gene is associated with an increased risk of esophageal squamous cell carcinoma (ESCC) [22]. In European countries, rs3200401 did not associate with GC and melanoma risk. However, the results from these studies were inconsistent. Thus, we pooled current published studies and conducted a meta-analysis to explore the potential relationships between *MALAT1* rs3200401 C > T and the risk of cancer.

## 2. Materials and Methods

This meta-analysis was conducted following the Preferred Reporting Items for Systematic Reviews and Meta-Analysis (PRISMA) Statement.

### 2.1. Search Strategy

A comprehensive search was performed on online databases, including PubMed, EMBASE and Web of Science up to 1 September 2021. The following search terms were used in the databases: “*MALAT1*” AND “neoplasm OR cancer OR tumor OR neoplastic OR carcinoma OR adenocarcinomas OR malignancy OR malignancies OR neoplasia” AND “single nucleotide polymorphism OR SNP OR variant OR variation OR polymorphism”. We have also carefully screened references of relevant publications to obtain potential studies.

### 2.2. Inclusion and Exclusion Criteria

Publications obtained through the search meet the following inclusion criteria: (1) the relationship between SNP rs3200401 C > T and cancer risk was described in case–control studies; (2) odd ratios (ORs) and 95% confidence intervals (CIs) can be estimated via the frequencies of genotypes or alleles; (3) the publications were in English only; (4) the data of the control group satisfied the Hardy–Weinberg equilibrium (HWE). The exclusion criteria for publications are as follows: (1) reviews, letters, case reports; (2) duplicate publications; (3) studies without sufficient or qualified data. In addition, two authors (K. Li and Z. Han) independently checked the relevant articles to assess whether studies met the criteria.

### 2.3. Data Extraction and Quality Assessment

The data of included studies were extracted by two independent reviewers (K. Li and Z. Han) according to the following contents: first author, publication year, region, ethnicity of study population, source of control, cancer type, genotypes of rs3200401 in case and control group, *p* value of HWE and genotyping methods. If the results of two reviewers were inconsistent, consensus was reached through discussion with the third reviewer (J. Wu). The Newcastle–Ottawa quality assessment scale (NOS) was used to assess the quality of included studies [23]. We rated the articles as 0–9 according to NOS: a score of six or above was considered to be a high-quality study and a score of four to five was considered as a medium-quality study [24].

### 2.4. Statistical Analysis

The indictors of ORs and 95%CI were used to assess the relationship between *MALAT1* rs3200401 C > T and cancer risk. Five genetic models (allelic, heterozygote, homozygote, dominant and recessive model) were applied for the analyses. To assess the heterogeneity of all studies, the Q-test and I^2^ test was performed. If the results showed I^2^ > 50% or *p* < 0.05, we would consider the heterogeneity to be significant and use a random-effect model (the DerSimonian–Laird method). If not, a fixed-effect model (Mantel–Haenszel method) would be used. Subgroup analysis of the quality of studies, type of cancer, ethnicity and source of control were performed to explore potential associations. Based on deleting medium-quality studies, sensitivity analysis was performed to assess the stability of the results by observing the alteration after excluding every single study in turn. Publication bias was evaluated using Begg’s funnel plot and Egger’s regression test. Meta-regression analysis was used to identify whether some factors were the source of heterogeneity. Trial sequential analysis (TSA) was carried out to assess the reliability of the results. The TSA parameter was set to a power of 80%, type I error of 5%, relative risk reduction of 15% and control event proportion of an average of each included study. STATA software version 15.1 (Stata Corporation, College Station, TX, USA) and TSA software version 0.9.5.10 (Copenhagen Trial Unit, Centre for Clinical Intervention Research, Rigshospitalet, Copenhagen, Denmark) were applied for statistical analysis.

## 3. Results

### 3.1. Characteristics of Studies

**Figure 1:**
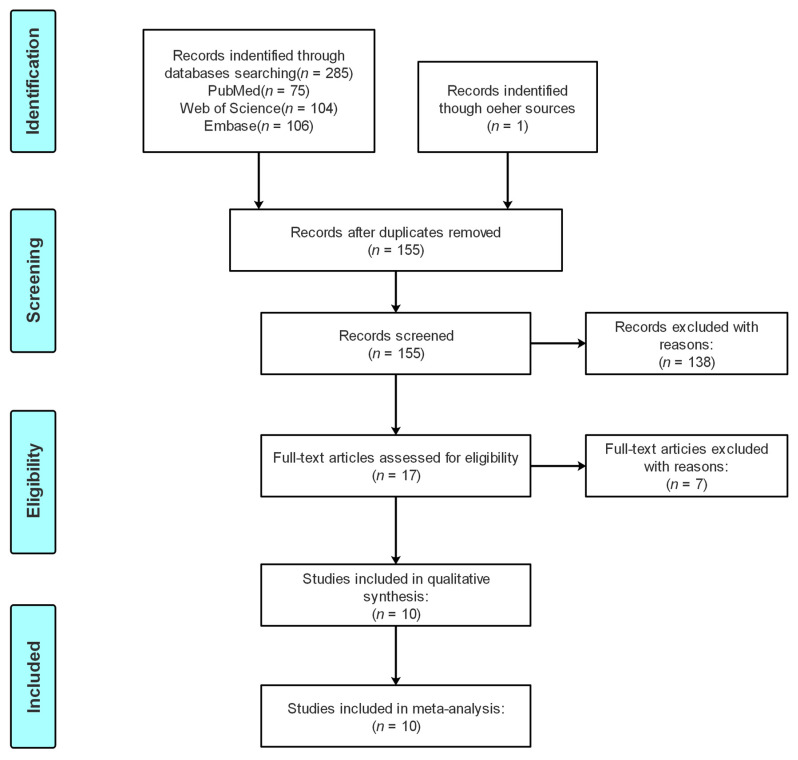
Flow diagram of selection studies in this meta-analysis.

The screening procedure is shown in Figure 1. We retrieved 285 potentially relevant publications from PubMed, Embase and Web of Science, and obtained one publication from references cited in the literature. Then, 269 irrelevant publications were discarded by screening titles and abstracts. Based on the exclusion criteria, we excluded seven publications after the full-text review. Finally, A total of 10 case–control studies fulfilling the inclusion with 6630 cases and 7457 controls criteria were included in this meta-analysis. The baseline characteristics of these studies are shown in Table 1 [19,20,21,22,25,26,27,28,29,30]. Among them, two studies investigated hepatocellular carcinoma, two studies investigated gastric cancer, two studies investigated colorectal cancer and four studies investigated other cancers (oral squamous cell carcinoma, melanoma, esophageal squamous cell carcinoma, papillary thyroid cancer). As for ethnicity, eight studies were conducted on Asians and two were on Caucasians. Control sources of four studies were population based, the others were hospital based. Most of the studies included in this meta-analysis were considered to be of high quality, and two studies scored less than 6. Table 2 showed the genotype frequency distributions and HWE of included studies. 

### 3.2. Quantitative Analysis

**Figure 2:**
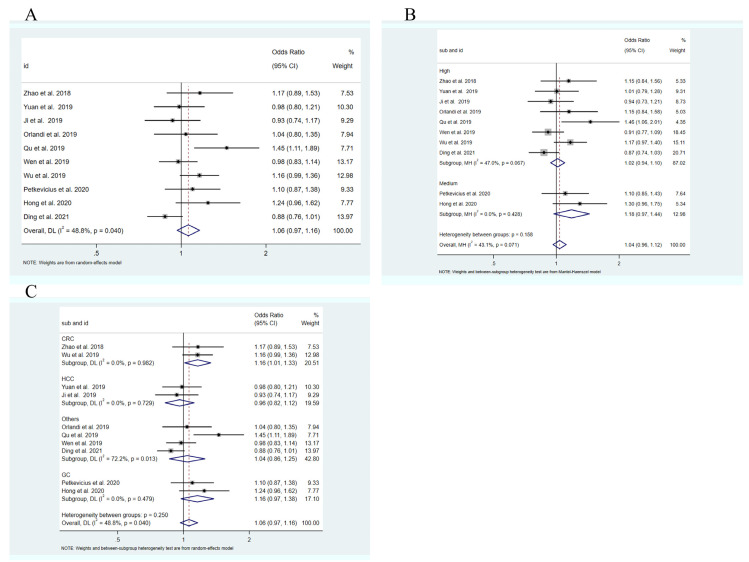
Forest plots of the relationships between rs3200401 and cancer risk. (**A**) Allelic model of rs3200401 (C vs. T). (**B**) Dominant model of rs3200401 for quality of studies (CC vs. CT + TT). (**C**) Allelic model of rs3200401 for cancer types (C vs. T).

The main results of the heterogeneity tests for five genetic models are presented in Table 3. For allelic genetic models, significant statistical heterogeneity was found (C vs. T: I^2^ = 48.8%, *p* = 0.040). Therefore, the combined data were calculated with a random-effect model in allelic models, and a fixed-effect model was used in heterozygote, homozygote, dominant and recessive models. The results of crude analysis indicated no significant association between rs3200401 C > T and cancer risk in all genetic models (Table 2, Figure 2A). In a subgroup analysis of study quality, rs3200401 C > T still was not significantly associated with cancer risk in the high-quality studies group (Figure 2B). Subgroup analysis was further conducted according to cancer type, ethnicity and source of control (Table 3). There was no significant evidence of the correlation that was found in ethnicity and control source subgroups. We also observed that the groups of HCC, GC and other cancers were not significantly related with rs3200401 C > T, while, in the CRC group, rs3200401 C > T increased the cancer risk in the allelic model (OR:1.16, 95%CI:1.01–1.33; Figure 2C).

### 3.3. Sensitivity Analysis and Publication Bias

**Figure 3:**
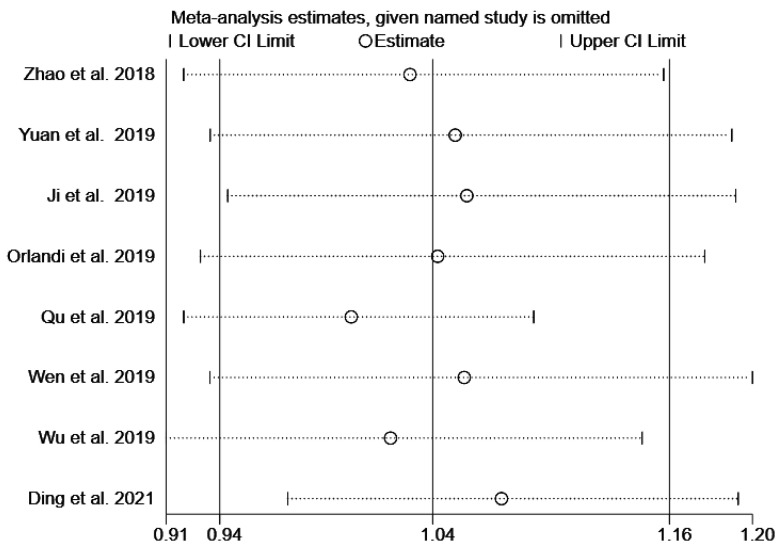
Sensitivity analysis of the influence in allelic model (C vs. T).

**Figure 4:**
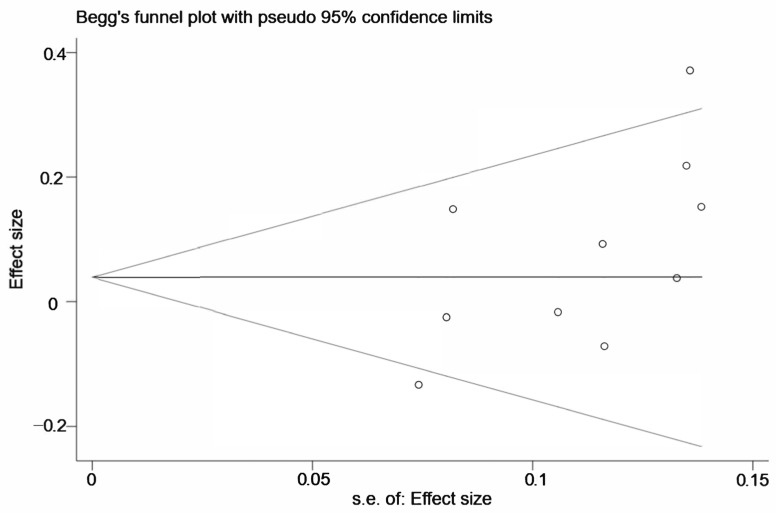
Begg’s funnel plot for publication bias test in allelic model (C vs. T). (s.e.: selogOR).

**Figure 5:**
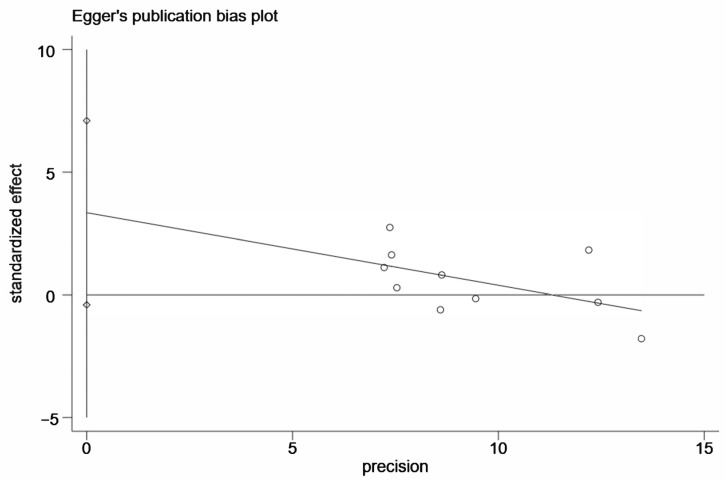
Egger’s test for publication bias test in allelic model (C vs. T).

Sensitivity analysis was conducted to evaluated whether the results were stable. We removed the eight high-quality studies in sequence and found no significant alteration, which indicated that the results were robust after the exclusion of medium-quality studies (Figure 3). We conducted Begg’s funnel plot and Egger’s regression test to evaluate the publication bias of the current meta-analysis. In Figure 4, the shape of Begg’s funnel plot was generally symmetrical, indicating no significant publication bias. The result of Egger’s test also confirmed no evidence of publication bias (*p* = 0.074, Figure 5). 

### 3.4. Meta-Regression Analysis

**Figure 6:**
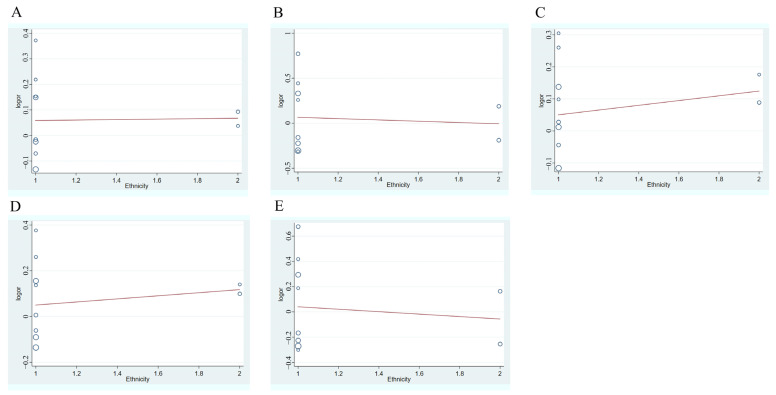
Meta-regression analysis of ethnicity in five genetics models. (**A**) Allelic (C vs. T); (**B**) Homozygote (CC vs.TT); (**C**) Heterozygote (CC vs. CT); (**D**) Dominant (CC vs. CT + TT); (**E**) Recessive (CC + CT vs. TT).

Because of heterogeneity in the quantitative analysis, a meta-regression analysis was conducted to explore potential sources of heterogeneity. To explore potential sources of heterogeneity, meta-regression was performed for covariables (ethnicity, source of control, study quality) in turn. The results suggest that ethnicity, source of control and quality of study were not statistically confirmed as confounding factors (Table 4, Figure 6). 

### 3.5. Trial Sequential Analysis

**Figure 7:**
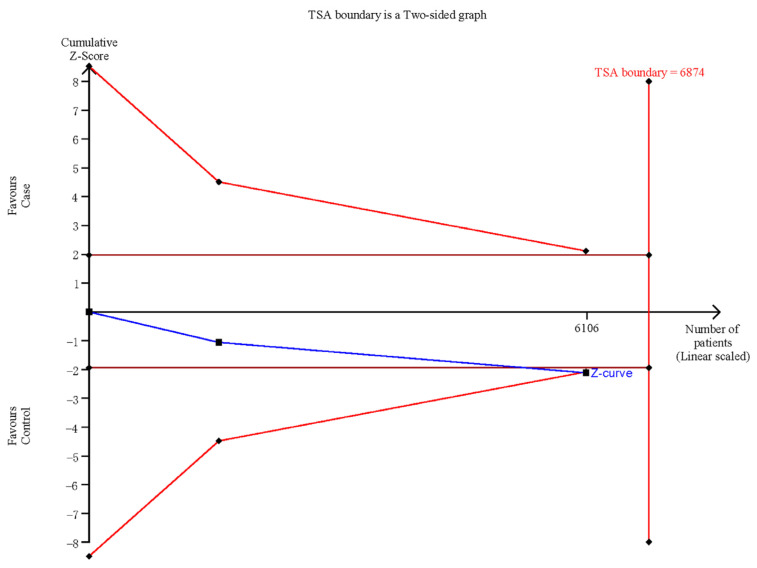
TSA for rs3200401 polymorphism and CRC risk in the allelic model.

We performed a trial sequential analysis to reduce the random errors and strengthen the robustness of the association between rs3200401 C > T and CRC risk. As shown in Figure 7, although, the cumulative Z-curve did not reach the required information size boundary, it crossed the traditional boundary and the TSA boundary. The result indicated that the cumulative evidence for the association is sufficient.

## 4. Discussion

Recently, many studies have focused on the relationship between genetic variation in *MALAT1* and cancer risk. *MALAT1* rs3200401 C > T polymorphism has also been extensively studied in relation to different cancer risks. However, the results of these studies have been inconsistent. We performed a meta-analysis to identify the role of rs3200401 C > T in cancer susceptibility. The crude analysis results showed no association between rs3200401 C > T and cancer risk in the five genetic models. We observed that Asians or Caucasians had no significant effect on the correlation between rs3200401 C > T and cancer susceptibility, suggesting that ethnicity is not a potential confounding factor. However, the current meta-analysis included only two European studies, and more studies are needed to confirm the results. Subgroup analysis by cancer type revealed that rs3200401 C > T increased the risk of CRC in the allelic model.

*MALAT1* is enriched in nuclear spots and influences the distribution of serine/arginine (SR) family splicing factors in the region. SR proteins can regulate the alternative splicing (AS) of pre-mRNA and alter the susceptibility of cancers [31]. rs3200401 C > T polymorphism is one of the binding sites of *MALAT1* to SRSF2. The mutation of rs3200401 may lead to the downregulation of SRSF2 phosphorylation and a change in tumor-related gene shearing, thus affecting the occurrence and development of cancer [32]. Hong et al. found that rs3200401 C > T was significantly associated with an increased risk of GC in men, especially intestinal-type GC after the stratification of patients [30]. The association between the risk of precancerous lesions of GC and rs3200401 C > T was reported by Vytenis et al. [29]. However, we did not find that rs3200401 C > T was associated with GC. The reason may be that few studies on GC and differences in the study population lead to no obvious association with GC. *MALAT1* was widely reported to be overexpressed in HCC patients [33,34], which promoted HCC cell proliferation, migration and invasion [35]. In our study, we observed that HCC risk had no association with rs3200401 C > T. Whether rs3200401 C > T polymorphism affected *MALAT1* expression in HCC needs to be further investigated. The results of the subgroup analysis showed that rs3200401 T allele was significantly associated with increased CRC risk compared with C allele. One study reported a tendency that serum *MALAT1* expression levels of the TT genotype and CT + TT genotype were higher than that of the CC genotype in CRC patients [36]. A similar trend was found in serum *MALAT1* expression levels between rs3200401 C>T and cerebral ischemic stroke [37]. Li et al. found that *MALAT1* mRNA was overexpressed in CRC tissues according to the Oncomine expression profiling database [38]. The upregulation of *MALAT1* expression promotes the development, invasion and metastasis of CRC through multiple pathways and is associated with poor prognosis [39,40]. Lampropoulou et al. showed that the rs3200401 CT + TT genotype was related with a significantly lower overall survival [41]. These studies suggested that the variation of rs3200401 may be related to the occurrence of CRC. In addition, the contribution of rs3200401 C > T polymorphism to the development of other cancers has been inconsistent. In lung adenocarcinoma, advanced lung adenocarcinoma patients with the CT + TT genotype had significantly longer median survival times compared with the CC genotype [32]. We considered that the T allele may be protective against cancer risk in adenocarcinomas compared with the C allele. Qu et al. reported that rs3200401 C > T was significantly associated with an increased risk of ESCC, and the same results were found in a subgroup of never drinking [22]. Despite the rs3200401 TT and CT + TT genotypes exhibiting a lower risk of OSCC, patients who carried T allele were more likely to develop high-grade OSCC in the subgroup of betel quid chewers [19]. Therefore, environmental carcinogens may affect the role of rs3200401 C > T polymorphism in the development of squamous cell carcinoma.

This is the first meta-analysis to assess rs3200401 C > T polymorphism associated with cancer risk to our knowledge. The included studies were conducted in recent years, which reduced heterogeneity to some extent. We performed a relatively comprehensive subgroup analysis to explore potential heterogeneity. There were also several limitations in our study. Firstly, it is insufficient because of the case–control studies enrolled in our study. Studies involving CRC are limited, and more studies of larger sample sizes are needed to verify the association between rs3200401 C > T polymorphism and CRC risk Secondly, we only retrieved the studies published in English. This may have caused us to ignore some studies published in other languages. Finally, due to the limited inclusion of studies, studies with low NOS scores were not excluded.

## 5. Conclusions

In summary, the study indicated that the association between rs3200401 C > T and overall cancer risk was not significant. However, rs3200401 C > T may increase the risk of CRC. Larger sample size studies on a wide range of cancer types are required to perform further verification about the relationship between *MALAT1* polymorphism rs3200401 and cancer risk.

## Figures and Tables

**Table 1:** The basic characteristics of the enrolled studies.

First Author	Year	Region	Ethnicity	Source of Controls	Type of Cancer	Sample Size (Case/Control)	Genotyping Method	NOS
Zhao	2018	China	Asian	HB	CRC	400/400	TaqMan	7
Yuan	2019	Taiwan	Asian	PB	HCC	394/1199	TaqMan	7
Ji	2019	China	Asian	HB	HCC	624/618	TaqMan	7
Orlandi	2019	Italy	Caucasian	PB	melanoma	334/291	PCR-RFLP	6
Qu	2019	China	Asian	HB	ESCC	245/490	TaqMan	7
Wen	2019	China	Asian	HB	PTC	140/100	TaqMan	7
Wu	2019	China	Asian	PB	CRC	1078/1175	TaqMan	7
Petkevicius	2020	Lithuania	Caucasian	HB	GC	613/476	TaqMan	5
Hong	2020	Korea	Asian	HB	GC	1134/1228	TaqMan	5
Ding	2021	Taiwan	Asian	PB	OSCC	1350/1199	TaqMan	8

Abbreviations: PB, population based; HB, hospital based; HCC, hepatocellular carcinoma; OSCC, oral squamous cell carcinoma; ESCC, esophageal squamous cell carcinoma; GC, gastric cancer; PTC, papillary thyroid cancer; CRC, colorectal cancer; PCR-RFLP: polymerase chain reaction–restriction fragment length polymorphism.

**Table 2:** Distribution of genotype and HWE of the *MALAT1* rs3200401 polymorphism.

First Author	Case			Control			HWE
	CC	CT	TT	CC	CT	TT	
Zhao	283	102	15	294	96	10	Y
Yuan	263	117	14	802	347	50	Y
Ji	464	149	9	453	152	12	Y
Orlandi	190	125	19	174	96	21	Y
Qu	148	79	18	338	133	19	Y
Wen	808	302	23	872	322	31	Y
Wu	751	294	33	856	292	27	Y
Petkevicius	416	171	21	335	126	14	Y
Hong	312	133	13	280	92	9	Y
Ding	948	363	39	807	347	45	Y

Notes: YES: *p*_HWE_ ≥ 0.05, NO: *p*_HWE_ < 0.05.

**Table 3:** Results of the meta-analysis from different genetic models.

Rs3200401	N	Allelic Model (C vs. T)				Homozygote Model (CC vs. TT)				Heterozygote Model (CC vs. CT)				Dominant Model (CC vs. CT + TT)				Recessive Model (CC + CT vs. TT)			
		*I* ^2^	*p*	OR (95% CI)	*p_Z_*	*I* ^2^	*p*	OR (95% CI)	*p_Z_*	*I* ^2^	*p*	OR (95% CI)	*p_Z_*	*I* ^2^	*p*	OR (95% CI)	*p_Z_*	*I* ^2^	*p*	OR (95% CI)	*p_Z_*
Overall	10	48.8%	0.040	1.06 (0.97–1.16)	0.215	24.0%	0.222	1.03 (0.84–1.25)	0.795	6.4%	0.382	1.06 (0.98–1.14)	0.167	43.1%	0.071	1.04 (0.96–1.12)	0.322	11.3%	0.339	1.01 (0.83–1.22)	0.951
Cancer type																					
CRC	2	0.0%	0.982	1.16 (1.01–1.33)	0.033	0.0%	0.820	1.44 (0.93–2.23)	0.103	0.0%	0.838	1.14 (0.97–1.34)	0.125	0.0%	0.918	1.16 (0.99–1.36)	0.060	0.0%	0.801	1.39 (0.90–2.15)	0.137
HCC	2	0.0%	0.729	0.96 (0.82–1.12)	0.605	0.0%	0.777	0.81 (0.49–1.34)	0.414	0.0%	0.698	0.99 (0.83–1.19)	0.941	0.0%	0.706	0.97 (0.82–1.16)	0.772	0.0%	0.804	0.81 (0.49,1.33)	0.407
Others	4	72.2%	0.013	1.04 (0.86–1.25)	0.096	60.1%	0.057	0.98 (0.62–1.53)	0.918	48.7%	0.119	1.01 (0.90,1.13)	0.872	67.7%	0.026	1.03 (0.84,1.27)	0.746	52.1%	0.100	0.95 (0.63,1.42)	0.789
GC	2	0.0%	0.479	1.16 (0.97–1.38)	0.096	0.0%	0.901	1.24 (0.72–2.13)	0.432	0.0%	0.415	1.18 (0.96–1.44)	0.118	0.0%	0.428	1.18 (0.97–1.44)	0.094	0.0%	0.965	1.19 (0.70,2.04)	0.527
Source of control																					
HB	6	46.3%	0.097	1.11 (0.98-1.26)	0.113	25.2%	0.245	1.17 (0.88-1.56)	0.281	0.0%	0.494	1.09 (0.98,1.21)	0.129	47.3%	0.091	1.06 (0.93,1.18)	0.261	12.0%	0.339	1.14 (0.85,1.52)	0.378
PB	4	54.5%	0.086	1.01 (0.88–1.15)	0.943	16.5%	0.309	0.92 (0.70–1.20)	0.526	35.4%	0.200	1.02 (0.92,1.14)	0.665	49.8%	0.113	1.01 (0.91,1.13)	0.786	3.5%	0.375	0.91 (0.70,1.18)	0.469
Ethnicity																					
Asian	8	59.7%	0.015	1.06 (0.95–1.19)	0.437	37.6%	0.129	1.03 (0.83–1.28)	0.762	22.1%	0.254	1.04 (0.96,1.13)	0.300	53.7%	0.035	1.05 (0.93,1.19)	0.392	24.6%	0.233	1.02 (0.82–1.26)	0.858
Caucasian	2	0.0%	0.753	1.07 (0.90–1.27)	0.428	0.0%	0.437	0.99 (0.62–1.59)	0.971	0.0%	0.693	1.13 (0.92–1.40)	0.253	0.0%	0.845	1.12 (0.92–1.37)	0.261	0.0%	0.383	0.94 (0.59–1.50)	0.807
Study quality																					
High	8	54.5%	0.032	1.04 (0.94,1.16)	0.449	38.1%	0.126	1.00 (0.81–1.23)	0.976	8.9%	0.361	1.04 (0.95,1.13)	0.392	47.0%	0.067	1.02 (0.94,1.10)	0.695	28.0%	0.205	0.98 (0.80,1.21)	0.855
Medium	2	0.0%	0.479	1.16 (0.97–1.38)	0.096	0.0%	0.901	1.24 (0.72–2.13)	0.432	0.0%	0.415	1.18 (0.96–1.44)	0.118	0.0%	0.428	1.18 (0.97–1.44)	0.094	0.0%	0.965	1.19 (0.70,2.04)	0.527

Abbreviations: N, number of studies; *p*, *p* value of Q-test for heterogeneity test; *p_Z_*, *p* value of Z-test for association.

**Table 4:** Meta-regression analysis of association between rs3200401 and the cancer risk.

SNP	Allelic Model		Homozygote Model		Heterozygote Model		Dominant Model		Recessive Model	
	Coef (95% CI)	*p*	Coef (95% CI)	*p*	Coef (95% CI)	*p*	Coef (95% CI)	*p*	Coef (95% CI)	*p*
rs3200401	Ethnicity									
	0.01 (−0.29,0.31)	0.947	−0.07 (−0.82,0.67)	0.830	0.07 (−0.21,0.36)	0.569	0.07 (−0.26,0.40)	0.649	−0.01 (−0.79,0.60)	0.755
	Source of control									
	−0.10 (−0.32,0.12)	0.337	−0.26 (−0.81,0.30)	0.316	−0.06 (−0.27,0.15)	0.521	−0.06 (−0.31,0.19)	0.586	−0.24 (−0.75,0.28)	0.318
	Study quality									
	0.11 (−0.18,0.40)	0.402	0.20 (−0.59,0.99)	0.577	0.12 (−0.15,0.40)	0.330	0.14 (−0.18,0.45)	0.343	0.18 (−0.56,0.92)	0.592

## Data Availability

The data supporting the findings of this study are available within the article.
